# Metabolism in Tumour-Induced Bone Disease

**DOI:** 10.1007/s11914-026-00956-3

**Published:** 2026-02-16

**Authors:** Renee T. Ormsby, Claire M. Edwards

**Affiliations:** 1 Nuffield Department of Surgical Sciences, University of Oxford, Oxford, UK; 2 Nuffield Department of Orthopaedics, Rheumatology & Musculoskeletal Sciences, University of Oxford, Oxford, UK

**Keywords:** Cancer metabolism, Bone metastasis, Multiple myeloma, Prostate cancer, Breast cancer, Bone disease

## Abstract

**Purpose of Review:**

This review highlights recent studies that investigate the metabolic reprogramming that occurs in specific types of bone metastatic cancers, including different types of breast cancer, prostate cancer and multiple myeloma.

**Recent Findings:**

Metastatic cancer cells use altered metabolic pathways to adapt in alternate environments. Cancer cells that home towards the bone are able to manipulate their metabolism to survive in the hypoxic microenvironment, shifting between oxidative phosphorylation and glycolysis, whilst also exploiting neighbouring cells within the bone to provide energy and protect against chemotherapies.

**Summary:**

Targeting the altered metabolic pathways in cancer cells could be used to improve treatments, reduce tumour burden and prevent the destruction of the bone that occurs in tumour-induced bone disease.

## Introduction

Metabolism is now considered a hallmark of cancer [1]. Disseminated cancer cells can adapt by altering their cellular phenotype and activity in order to survive in a different environment from their origins [2]. This adaptation includes altering the metabolic pathways within the cell to obtain enough energy to survive outside of the primary tissue. Metastatic cancer cells modify their metabolism to invade the bone, surviving in low oxygen conditions. Cancer cells then activate a vicious cycle of bone destruction, destabilizing bone homeostasis to promote resorption, causing the formation of osteolytic lesions. This ultimately causes both bone breakdown and absolute invasion by the tumour. Alternatively, cancer cells form osteoblastic lesions where newly formed bone disrupts the normal bone microenvironment, engulfing the bone marrow and expanding into the cortex, compromising the structural integrity of the bone. Both osteolytic and osteoblastic lesions, or a combination of both, lead to tumour-induced bone disease. As a result, the tumour-induced bone disease causes alterations in the bone microstructure, compromising the strength of the bone and leading to fractures, increased pain and increased mortality. The tumour-induced bone disease then feeds back to promote tumour growth and survival, in part by impacting tumour metabolism, and ultimately establishing a vicious cycle of tumour growth and bone disease.

The bone microenvironment is a key component contributing to the shift in metabolism in metastatic cancer cells that home to the bone. Multiple factors influence this, including the decrease in oxygen leading to highly hypoxic areas within the bone, the presence of reactive oxygen species which are key for bone metabolic pathways, and importantly, the multi-cellularity of the bone marrow. Bone-residing cells such as stromal cells, osteoblasts and osteoclasts have been shown to contribute to cancer progression within the bone, providing growth factors and metabolites that allow cancer cells to thrive and survive even after treatment [3]. This cellular crosstalk indicates that both bone cells and cancer cells contribute to bone destruction and tumour growth.

This review will discuss metabolic pathways recently investigated in cancers associated with tumour-induced bone disease, including breast cancer, prostate cancer and multiple myeloma. We will discuss how cancer cells are able to shift from oxidative phosphorylation to glycolysis, in a process termed the Warburg effect, and in certain cancers switch back, adapting to the environment and energy demands of the cell. This review will highlight the findings that focus on how cancer cells alter their metabolism to disseminate and adapt within the bone microenvironment, and how bone cells contribute to the metabolic dysregulation driving cancer cell survival and tumour-induced bone destruction.

## The Bone Microenvironment and Tumour Cells

Metastatic cells extravasate into the circulation through an epithelial to mesenchymal transition (EMT), a key process which allows cancer cells to become more migratory and invasive, with an increased resistance to apoptosis [4]. Circulating cancer cells then home to the bone, directed by chemokines produced by bone cells which induce chemotaxis. Once in the bone, the microenvironment provides a fertile soil, as originally described by Paget [5], due to both the abundant growth factors and the mineralized extracellular matrix which inadvertently support cancer growth. Metastatic cancer cells from breast cancer, prostate cancer and lung cancers are known to metastasize and form tumours within the bone, inducing bone disease.

As mentioned, the bone microenvironment is comprised of multiple cell types including cells which maintain bone homeostasis. These include bone forming cells, osteoblasts, bone resorbing cells, osteoclasts, and bone embedded cells, osteocytes. Dysregulation of these cells, such as excessive activation of osteoclasts and bone resorption is a key feature involved in tumour-induced bone disease. The bone marrow, importantly, is the site of haematopoiesis and contains a plethora of cells including haematopoietic stem cells, mesenchymal stem cells, which differentiate into bone marrow stromal cells, macrophages, adipocytes, endothelial cells and immune cells. Many of these cell types contribute to cancer survival within the bone and influence tumour-induced bone disease.

Mesenchymal stem cells (MSC) present within the bone marrow are multipotent cells which contribute to bone formation, fracture repair and bone regeneration, as well as modulating immune responses [6]. MSCs can differentiate into a variety of cell types including bone marrow stromal cells and osteoblasts, as well as fibroblasts, adipocytes and chondrocytes. MSCs have been shown to alter the metabolic profile of cancer cells, reducing reactive oxygen species and promoting a shift towards glycolysis [7, 8]. Studies have shown breast cancer cells that have metastasized to the bone undergo metabolic reprogramming when in the presence of MSCs. Estrogen-positive breast cancer cells (ER+ BC) in contact with MSCs shifted towards oxidative phosphorylation, with increased ATP, which caused an overall resistance to chemotherapies [9]. A recent study has shown MCT4, a lactate transporter, was upregulated in ER+ BC cells cocultured with MSCs. Treatment with syrosingopine, which binds to and inhibits the function of both MCT1 and MCT4, prevented lactate and proton transport from the cell, and reduced intracellular ATP in the cancer cells [10]. Co-cultured ER + BC cells and MSC cells were then treated with both syrosingopine and the selective estrogen degrading drug, fulvestrant. Combined treatment led to a decrease in cancer cell viability by reducing resistance to fulvestrant.

As mentioned above, MSCs can differentiate into bone marrow stromal cells (BMSCs), which are involved in cancer homing to the bone and their subsequent survival and proliferation, in part by altering cancer cell metabolism [11]. Stromal cells and osteoblasts produce key chemokines which attract cancer cells to the bone [12]. The production of CXCL12, or stromal cell-derived factor 1 (SDF-1) by stromal cells and osteoblasts, was discovered in the early 2000's and was shown to be a crucial chemoattractant that drives cancer cell homing and adhesion to the bone [13]. The corresponding chemokine receptor, CXCR4, is produced by many cancer types including prostate, breast, brain, lung and melanoma. The CXC12-CXCR4 axis is crucial for cell migration involved in vascularization and organ homoeostasis. Once extravasated into the bone microenvironment, cancer cells hijack the normal pathways that modulate bone homeostasis.

Bone marrow stromal cells have been shown to support prostate cancer (PCa) growth in the bone by upregulating the pentose phosphate pathway. PCa cells utilize this branch of glycolytic pathway to provide key amino acids and regulate their cellular redox state in order to combat the reactive oxygen species present in the bone. We have shown non-bone metastatic PCa cells (LNCaP), alter their metabolism in the presence of BMSCs by upregulating the pentose phosphate pathway, in particular by increasing G6PD, the rate limiting enzyme which regulates the conversion of NADP+ to NADPH in the cell [14]. Increased G6PD is associated with increased antioxidants and decreased reactive oxygen species, allowing the cancer cells to adapt to the bone microenvironment.

Stromal cells have also been shown to support myeloma viability through the transfer of mitochondria. This process, described by Marlein et al*.*, illustrated intercellular mitochondrial transfer between myeloma cells and BMSCs via tunnelling nanotubes [15]. Mitochondria transferred from BMSCs to myeloma cells promoted oxidative phosphorylation. In a recent study by Matula et al*., *myeloma cells were shown to increase mitochondrial transfer in a bidirectional transfer mechanism in response to an increase in chemotherapeutics. The tunnelling nanotubes and partial cell fusion between cells allowed mitochondrial transfer from the myeloma cells to stromal cells. This occurred at increased rates when the cells were treated with chemotherapies, decreasing superoxide levels in myeloma cells, whilst increasing ATP levels and survival rates. This study used primary myeloma cells as well as corresponding stromal cells from individual patients and highlights the importance of targeting metabolism in cancer cells [16].

Bone marrow stromal cells have the capacity to differentiate into bone marrow adipocytes (BMAds), which are fat cells that function as energy stores. Cancer cells have been shown to push BMSCs towards adipogenesis over other cellular states of differentiation [17]. This leads to downstream effects including the suppression of osteogenic factors which promote bone formation, and the production of adipokines which directly and indirectly stimulate bone resorption, all of which has consequences regarding bone disease. Additionally, studies have shown the importance of BMAds in cancer metabolism.

BMAds are a primary source of lipids and cancer cells have been shown to induce delipidation of adipocytes, providing a fuel source that supports cancer growth within the bone microenvironment [18]. In myeloma, BMAds contribute to myeloma progression by providing growth factors, adipokines and adipocytokines that support MM proliferation, viability and migration. Myeloma cells in turn alter BMAds, such as downregulating adiponectin, a key adipokine which has anti-tumour effects. Myeloma cells were also shown to stimulate lipolysis in BMAds, reducing the size of lipid droplets in the BMAds [19], and stimulating the release of free fatty acids and upregulating fatty acid transporters [20]. An interesting finding from this study showed BMSCs from patients with either MGUS or smouldering multiple myeloma had an increased commitment towards adipogenesis. This suggests BMSCs within the bone are preconditioned towards adipogenesis in both the precursor and early stages of myeloma, which would have consequences for bone homeostasis, with an associated reduction in osteogenesis and bone formation. In fact, altered BMAds were shown to produce key adipogenic signalling molecules such as visfatin [21], which were upregulated in MM and indirectly stimulate bone resorption by upregulating pro-osteoclastic factors such as IL-6 and matrix metalloproteinases [22].

Another study has shown the interaction between adipocytes and MM cells may have implications for treatment response. MM cells were shown to stimulate genes associated with senescence in adipocytes, including increased IL-6, IL-8, and MCP-1, whilst reducing lipid content and altering the expression of genes associated with adipocyte function, such as RUNX2, ADIPOQ and FABP4 [23]. Senescent BMAds likely contribute to the increase in MM proliferation and could indicate that MM cells are reprogramming BMAds to support MM survival within the bone. Importantly, this study showed senolytic treatments *in vitro* led to improved sensitivity to treatments with dexamethasone, a common treatment for MM, reducing tumour number after 72 h.

Adipocytes can also support prostate cancer (PCa) proliferation and invasion by supplying cancer cells with lipids, increasing lipid transporters and lipid uptake. Adipocytes also activate hypoxia signalling, and increase antioxidant production, all of which supports PCa metastasis, adaptation and survival within the bone microenvironment. A recent study by Herroon et al*.* investigated the transcriptome of bone-metastatic PCa cells, identifying the upregulation of endoplasmic reticulum (ER) stress and unfolded protein response (UPR) genes by bone marrow derived adipocytes in two different PCa cell lines [24]. Several genes were also found to be upregulated in data from patients with metastatic PCa. Both ER stress and UPR are key for cancer cell survival within the bone, thus adipocytes support PCa survival by stimulating lipid metabolism and activating adaptive signalling which allows the cells to survive within the bone microenvironment.

Bone resorbing osteoclasts are also involved in cancer progression and play essential roles in cancer cell invasion. Osteoclasts are stimulated by secreted factors from cancer cells including tumour necrosis factor-alpha (TNFα), PTHrP, IL-6, IL-8 and IL-11, and key osteoclastogenic factors, M-CSF and RANKL. Activated osteoclasts resorb the bone, releasing growth factors such as transforming growth factor-b (TGFβ), platelet derived growth factor (PDGF), bone morphogenic proteins (BMPs) and vascular endothelial growth factor (VEGF) [25], which stimulate tumour growth and bone destruction. This bi-directional signalling is referred to as the vicious cycle of bone metastasis, where osteoclasts indirectly stimulate cancer cell growth within the bone.

Stressed cancer cells rely on glutamine present in the microenvironment as an energy source. A recent study suggests osteoclasts are capable of providing breast cancer cells (BCa) with glutamine, upregulating glutathione metabolism, which aids in neutralizing the reactive oxidative species prevalent in the bone microenvironment. This study showed inhibition of osteoclasts with zoledronate, a bisphosphonate which binds to osteoclasts, led to a decrease in the glutamine cytosolic pathway and prevented BCa cell survival when challenged with cisplatin and the PARP inhibitor, olaparib. Combining zoledronate and the PARP inhibitor reduced the production of glutamine by osteoclasts and was synergistic towards reducing bone metastasis in a murine model of bone metastatic breast cancer [26].

Cancer cells within the bone microenvironment rely on surrounding cells to support cancer growth and viability. By altering cells within the tumour microenvironment, cancer cells are able to suppress anti-tumour responses, are protected from the effects of chemotherapies, and form vital interdependent metabolic relationships. One consequence of this is the adaptation of stromal cells into tumour-associated stromal cells (TASC) [27], which include fibroblasts, mesenchymal stem cells, adipocytes and endothelial cells. These reprogrammed stromal cells have altered cellular profiles; as mentioned above, MM cells can stimulate the production of pro-resorptive factors in altered adipocytes. Furthermore, a study has shown that MM cells induce lipolysis in adipocytes, whilst inducing senescence which leads to the production of senescence associated secreted proteins (IL-6, CXCL2), which can stimulate resorption by osteoclasts, causing osteolysis observed in multiple myeloma [23]. This study also induced senescence in adipocytes by irradiation; the senescent BMAds were then shown to protect MM cells from dexamethasone induced cell cycle arrest and apoptosis, in a similar manner to BMAds reprogrammed by MM cells.

## Oxidative Phosphorylation (OXPHOS)

Oxidative phosphorylation (OXPHOS) is the predominant metabolic pathway used by cells to generate ATP. OXPHOS occurs within the mitochondria of the cell, generating more than 95% of the cells energy (ATP). Metabolic reprogramming is now known to be an important factor required for cancer cells to adapt and maintain growth; part of this adaptation is the alteration of cancer cells from a reliance on OXPHOS to glycolysis. However, the variation in metabolic profile depends on not only the origins of the cancer type, but also modifications that can occur with treatment and the subsequent metastatic site. Studies have shown that breast cancer cells that are transitioning to the metastatic state display increases in both OXPHOS and glycolysis, but additionally, distinct metabolic changes that are associated with site-specific metastases.

One study investigated the metabolic alterations in breast cancer that produce calcifications, which is a clinical sign of increased malignancy of the cancer and is associated with metastasis [28]. Breast cancer cells (BCa) have been shown to undergo osteogenic differentiation and have increased expression of genes associated with osteoblast function and transcription, including RUNX2, ALP, BSP and BMP2, which control the production of calcium. Increased expression of these markers was associated with a decreased overall survival rate and lower metastasis-free survival. This study showed osteogenic differentiation caused an increase in OXPHOS, which then stimulated an epithelial to mesenchymal transition (EMT) [29]. Inhibition of OXPHOS in this model by using rotenone, an inhibitor that blocks the mitochondrial complex I and decreases ATP production, blocked the EMT transition in this model. This study highlights the impact of OXPHOS and mitochondrial activity in BCa and the influence this shift in metabolic reprogramming has on the metastatic potential of the cancer.

## Glycolysis

As mentioned, disseminated cancer cells extravasate into the bone microenvironment, where they encounter altered conditions that requires acclimatization in order to survive. A key feature of the bone microenvironment is the change in oxygen levels, with increased hypoxia prevalent within the bone marrow. Cancer cells which enter the bone have been shown to adapt by altering their metabolic pathways from oxidative phosphorylation to aerobic glycolysis, termed the Warburg effect. As mentioned above, cancer cells use glycolysis to rapidly proliferate, increasing glucose uptake at high rates.

A study has investigated the changes in breast cancer cells and a subsequent bone homing cell line variant. BCa cells were shown to rely heavily on glycolysis for energy metabolism, with only 20–25% of ATP produced by the mitochondria. Interestingly, the bone homing variant was shown to have a decreased demand for ATP, a lower oxidative stress and utilized glucose over glutamine as an energy source in comparison to the BCa parent cell line (MDA-MB-231). Additionally, this study described an increase in enzymes which synthesize serine and the serine transporter, SCL1A4, a factor known to stimulate osteoclastic bone resorption, contributing to tumour induced bone disease in BCa [30].

Alterations in the metabolic profile of prostate cells also contributes to metastasis. Prostate epithelial cells have a unique cellular metabolism; the cells have elevated levels of zinc levels which leads to an accumulation of citrate which is essential for normal prostate function. In comparison, metastatic PCa cells become more energy efficient, reducing the high levels of zinc and improving citrate oxidation through the tricarboxylic acid (TCA) cycle. This allows PCa cells to produce more ATP, becoming more energy efficient. We now know bone resident cells can also influence the shift in metabolism; bone marrow adipocytes have been shown to stimulate glycolysis in PCa cells in a bidirectional action, where BMAds stimulate the expression of glycolytic enzymes, production of lactate and OXPHOS by paracrine signalling [31]. Other bone residing cells have been shown to upregulate glycolysis in PCa. As mentioned above, bone marrow stromal cells also upregulate glycolysis and the pentose phosphate pathway in bone metastatic PCa. In addition, we have also shown glycolysis and the pentose phosphate pathway were upregulated in patients with bone metastatic PCa compared to patients with primary prostate cancer. Expression of G6PD, the rate limiting enzyme in the pentose phosphate pathway, correlated with increased disease severity, hormone sensitivity and cancer-free survival. Chemical inhibition of G6PD by 6-aminonicotinamide, an antimetabolite of nicotinamide, which mimics G6PD, has been shown to inhibit the function of G6PD and disrupt the pentose phosphate pathway. Bone metastatic PCa cells treated with 6-AN showed reduced cell viability, suggesting G6PD is key for PCa survival within the bone. Similarly, knockdown of G6PD (shG6PD) in the bone metastatic PCa cell line, PC3, lead to reduction in tumour growth in an intra-tibial murine model of PCa. The shG6PD-PC3 cells showed increased apoptosis, as well as increased ROS levels and decreased glutathione levels, which led to an increased sensitivity to chemotherapy. Therefore, targeting the pentose phosphate pathway in bone metastatic PCa is of interest, and could improve the efficacy of current chemotherapies through this altered metabolism.

Other subsets of prostate cancer have altered metabolism, including castration-resistant prostate cancer which results from resistance to androgen depletion therapy (ADT). This lethal form of PCa, termed androgen receptor-expressing PCa (ARPC), retains androgen receptor expression and continues to develop despite low circulating testosterone levels. A subtype of ARPC, “aggressive variant prostate cancer” (AVPC), has been identified, likely developing in response to chemotherapies, both of these forms of PCa have a high metastatic rate, disseminating predominantly towards the bone. A study by Mossa et al*., *describes the altered metabolic features of these two subtypes of castration-resistant PCa. Transcriptomic analysis of patient gene sets showed distinct differences in metabolic enrichment, with the ARPC relying on OXPHOS, compared to glycolysis in the AVPC. These results were also consistent in patient derived xenograft models [32].

## Lipid Metabolism

Alterations in lipid metabolism have been shown to be advantageous to cancer progression. Cancer cells have increased lipogenesis, increasing the production of biomolecules including lipoproteins, fatty acids, glycerides and non-glyceride lipids. Lipids are essential for the structural component of the cell membrane and contribute to the stabilization of the cell; lipids also act as signalling molecules and importantly, provide an energy source for the cell.

Cholesterol, in particular, is vital for cell proliferation as well as acting as an intracellular signal transducer. High cholesterol has been associated with an increased risk for cancer, as well as modulating anti-tumour immune cell responses. Comparatively, patients with multiple myeloma often have low levels of total cholesterol, low-density lipoprotein (LDL) and high-density lipo-protein (HDL) cholesterol leading to hypocholesterolaemia. Importantly, cholesterol in the blood stream is primarily in the form of low-density lipoprotein (LDL), and new research has shown myeloma cells alter their cholesterol metabolism to increase uptake of LDL. In a murine model of MM, increased dietary cholesterol promoted myeloma development and drug resistance, suggesting myeloma cells increase lipid uptake in order to survive [33].

A similar study has shown low-density lipoprotein receptor-related protein 8 (LRP8) is a key regulator of cholesterol metabolism in MM [34]. Over expression of LRP8 showed increased bone destruction in mice, with reduced BMI and trabecular thickness. Additionally, inhibition of LRP8 by lentiviral transduction (shLRP8) reduced cholesterol uptake by MM cells, likely caused by cell cycle arrest inducing autophagy in these modified cells. This study also showed the mTORC1 signalling pathway was activated by LRP8 with cholesterol uptake, and inhibition of LRP8 prevented MM cells from metabolizing cholesterol, inducing autophagy as a protective state.

## Summary

In summary, alterations in the metabolic state of cancer cells that disseminate to the bone are dependent on a variety of factors. Cancer cells are able to adapt and switch between metabolic pathways in order to survive and can rely heavily on unsuspecting neighbouring cells that co-exist in the tumour microenvironment, providing energy, at the risk of their own cellular state. Understanding how cancer cells shift between oxidative phosphorylation and glycolysis is key to developing and improving therapeutics that can accurately target cells; however, it is important to determine if other factors support cancer cell survival, such as lipid metabolism.

This review highlights the alterations that can occur not only in different cancers, but between different sub-types, such as oestrogen-receptor positive breast cancer and triple negative breast cancer, where the shift from glycolysis to oxidative phosphorylation could provide new effective drug targets that take advantage of the metabolic reprogramming that occurs in the cells, preventing tumour-induced bone disease and protecting the bone.

## Key References

- Buschhaus JM, Rajendran S, Chen S, Wharram BL, Bevoor AS, Cutter AC, et al. Bone marrow mesenchymal stem cells induce metabolic plasticity in estrogen receptor-positive breast cancer. Molecular cancer research: MCR. 2023 May 1;21(5):458.
  - ○ Uses single cell biosensors for metabolism and cocultures of breast cancer and mesenchymal stem cells to implicate the lactate transporter MCT4 in metabolic reprogramming and drug resistance
- Whitburn J, Rao SR, Morris EV, Tabata S, Hirayama A, Soga T, et al. Metabolic profiling of prostate cancer in skeletal microenvironments identifies G6PD as a key mediator of growth and survival. Sci Adv. 2022 Feb 25;8(8):eabf9096.
  - ○ Combines in silico analysis of patient datasets with metabolomic profiling of prostate-bone cocultures and in vivo study of prostate cancer bone metastasis to demonstrate metabolic plasticity of prostate cancer cells in bone and identify G6PD \as a potential metabolic target.
- Fan H, Xu Z, Yao K, Zheng B, Zhang Y, Wang X, et al. Osteoclast Cancer Cell Metabolic Cross-talk Confers PARP Inhibitor Resistance in Bone Metastatic Breast Cancer. Cancer Res. 2024 Feb 1;84(3):449–67.
  - ○ Use metabolic profiling to study interactions between osteoclasts and breast cancer cells and identify a role for GPX4 in PARP inhibitor resistance in breast cancer bone metastases.
- Jekabsons MB, Merrell M, Skubiz AG, Thornton N, Milasta S, Green D, et al. Breast cancer cells that preferentially metastasize to lung or bone are more glycolytic, synthesize serine at greater rates, and consume less ATP and NADPH than parent MDA-MB-231 cells. Cancer & Metabolism. 2023 Feb 20;11(1):4.
  - ○ Study the metabolic phenotype of bone metastatic breast cancer cells, revealing distinct metabolic changes.

## Data Availability

No datasets were generated or analysed during the current study.
